# The Skill of Positive Solitude Moderates the Relationship between 24 Character Strengths and Flourishing in the Second Half of Life

**DOI:** 10.3390/bs14090788

**Published:** 2024-09-09

**Authors:** Noa Bachman, Yuval Palgi, Ehud Bodner

**Affiliations:** 1 Department of Gerontology, University of Haifa, Haifa 3103301, Israel; ypalgi@research.haifa.ac.il; 2 Department of Social and Health Sciences, Bar-Ilan University, Ramat Gan 5290002, Israel; ehud.bodner@biu.ac.il; 3 Department of Music, Bar-Ilan University, Ramat Gan 5290002, Israel

**Keywords:** flourishing, loneliness, positive solitude, 24 character strengths, wellbeing

## Abstract

Objectives: Aging may challenge life and even affect individuals’ wellbeing and flourishing. This includes the challenges of diminished social connections and the experience of solitude in later life while seeking to leverage personal strengths. The current study examines two important personal resources, i.e., the skill of positive solitude and the 24 character strengths, which may be associated with flourishing in old age. Methods: A convenience sample of 1085 community-dwelling adults (M = 57.20, SD = 6.24, range = 50–87) completed an online survey with demographic measures and scales measuring personal strengths, the skill of positive solitude, and flourishing. Results: As hypothesized, the 24 character strengths and positive solitude were each associated with flourishing, and positive solitude moderated the relationships between the 24 character strengths and flourishing. The relationship between the 24 character strengths and flourishing was stronger among participants with lower levels of positive solitude. Conclusions: These findings highlight the way in which, despite the decrease in social relations which often characterizes old age, the skill of positive solitude serves as a valuable resource for flourishing in the later stages of life.

## 1. Introduction

Aging can inflict substantial life adversities, such as decreased social connections and social support, which may lead to loneliness [1] and undermine older adults’ wellbeing [2]. Alongside these age-related vulnerabilities, old age may contain strengths and flourishing [3,4]. Research has focused more on the weaknesses and dysfunctions of older adults than on their capabilities and strengths, and less is known about the personal resources that contribute to wellbeing during the second half of life [2,5]. Thus, the question arises regarding what personal resources one possesses that may help life thrive and flourish despite these adversities.

Character strengths are considered valuable personal resources that have been the focus of a growing body of literature in recent years [6]. Nevertheless, the skill of positive solitude differs and is not included among the 24 character strengths suggested by McGraph [7]. The skill of positive solitude is defined as a volitional inner process directed towards carving out time by oneself (without meaningful engagement with others) to become a valuable experience [8]. Its importance increases during the second half of life, as older adults are required to spend a substantial portion of their time in solitude. Both the 24 character strengths and the skill of positive solitude are separate capabilities that can enhance a person’s adjustment and may be associated with the ability to flourish despite potential adversities encountered during middle and old age. While the 24 character strengths focus on the positive aspects of personality that enable growth and development, positive solitude is the skill of enjoying time alone without significant engagement with others [9]. Moreover, both terms share some similar attributes, such as spirituality, creativity, and a sense of autonomy, which enable individuals to adapt and overcome stressful situations [9,10,11,12]. By adopting these strengths, older adults can enhance their capacity to flourish, even in solitude. Following this line of thought, this investigation aimed to delineate the relationships between 24 character strengths and flourishing in the second half of life and examine the moderating role of the skill of positive solitude in this relationship.

### 1.1. Flourishing

Flourishing is a state of overall wellbeing which is related to the presence of good mental health [13]. Flourishing complies with both the hedonic wellbeing approach, which concerns happiness and subjective wellbeing [14], as well as with eudemonic wellbeing, which involves the sense of the individual’s perspective on his/her life meaning. Although the hedonic and eudemonic approaches represent different perspectives towards wellbeing, wellbeing can also be defined as a multidimensional phenomenon, combining features from both approaches, which together form the basis for the concept of flourishing [15].

Flourishing is based on the premise that people thrive and flourish when they function well in their relationships, resulting in the experience of positive emotions. A thriving life is not only associated with good relationships but also with autonomy, a sense of self-efficacy, a sense of purpose, and positive feelings related to enjoyment, involvement, and satisfaction [16]. As flourishing encompasses a wide range of wellbeing, its attainment might be linked to one’s inherent personal strengths. In particular, character strengths, such as the 24 virtues in action (VIA), may significantly aid individuals in navigating the challenges of later life [17].

### 1.2. The 24 Character Strengths

One of the key elements of the school of positive psychology is the recognition of people’s inherent strengths, which are not always apparent and may need to be revealed [6]. According to positive psychology, each person possesses unique personal strengths, and recognizing these strengths enables individuals to flourish [6,17].

A central concept in positive psychology related to personality strengths is “good character” [7]. The 24 VIA classification [6], the most well-known model in this field, includes 24 character qualities which are considered as the positive aspects of personality [18]. These characteristics are arranged into six core virtues, each of which includes 3–6 strengths: wisdom and knowledge (comprising the virtues of creativity, curiosity, judgment, love of learning, and perspective); courage (comprising bravery, perseverance, honesty, and Zest); humanism (comprising love, kindness, and social intelligence); justice (comprising teamwork, fairness, and leadership); temperance (comprising forgiveness, humility, prudence, self-regulation), and transcendence (comprising appreciation of beauty and excellence, gratitude, hope, humor, spirituality) [6,17].

These strengths were characterized as good, stable, changeable, and ethically valued attributes, contributing to wellbeing. Research indicates that endorsing these 24 character strengths is associated with higher levels of thriving, including subjective, social, and psychological wellbeing [19,20,21,22].

Peterson and Seligman (2004) proposed that their classification of the 24 character strengths and virtues is related to the five-factor model (FFM) of personality [23,24], a connection supported by research [25]. However, fewer research efforts have been directed toward studies investigating the role of these strengths in the second half of life [17]. This gap is significant because, although strengths are aptitude-like, and are thus stable across situations and time, they remain potentially flexible, with each character strength exhibiting a different degree of flexibility [6,17]. Moreover, while the 24 character strengths have a biological and genetic basis [26] they can also play different roles when their integral effect is evaluated during the second half of life [6,17,27,28] and may even improve throughout life via rituals and practices tailored to individual tendencies and needs [6,17,29]. Given that people in the second half of life tend to spend an increasing proportion of their time alone [30], we suggest that the 24 character strengths can be better utilized when combined with the skill of positive solitude. More specifically, under the negative outcomes of solitude (e.g., loneliness), character strengths might not be utilized effectively as human resources. Nonetheless, the skill of positive solitude may improve one’s resources and enhance the utilization of the character strengths during the second half of life.

### 1.3. Positive Solitude

Positive solitude is a distinctive experience and a skill [9], defined as the ability to devote time to a meaningful experience taken by oneself, with or without the presence of others [8]. In addition, it has numerous distinctive features. At its core lies the ability to choose, which is grounded in a sense of autonomy, mastery, and competency—three characteristics that might develop over the life course [31,32]. Furthermore, because it is based on the freedom of choice, individuals may choose to experience positive solitude episodes even in the face of adversity.

Previous studies examining associations between solitude and social abilities, such as introversion, neuroticism, and sociability [33,34,35], have yielded inconsistent results [36]. Studies have suggested that, among adolescents and adults, the intrinsic motivation to spend time alone is linked with [37] a preference for solitude [33,37], neuroticism [33,38,39], and even a lack of sociability [40]. Nevertheless, recent studies [9,41,42] suggest that, when solitude is experienced voluntarily, it is associated with extroversion. These findings align with theories suggesting that, paradoxically, the ability to engage with others is closely related to the ability to be at ease in solitude [43]. According to Winnicott (1958), the capacity to be alone is not really opposed to the ability to be with others but is rather a complementary ability that produces the dialectic between being alone and being with others, which lasts throughout life. This ability, akin to secure attachment [44], allows individuals to engage socially when possible while feeling comfortable to be in solitude when they choose to. Additionally, by maintaining significant connections with others, individuals can experience being alone not as a state of loneliness but as a state of positive solitude. Conversely, when these complementary abilities to socialize and to be in solitude are lacking, solitude may lead to feelings of anxiety and alienation [45,46].

Positive solitude is associated with a wide range of potential benefits. These benefits may greatly enhance people’s emotional, psychological, and physical wellbeing [32,47]. As positive solitude may continue to develop in old age [31,32,35,48], it can be beneficial when considering the decrease in social connections during this stage of life. Embracing positive solitude in later years can provide numerous advantages and play a crucial role in fostering personal virtues tailored to their needs.

The skill of positive solitude encompasses various qualities that open new possibilities for older individuals, including fostering achievements, problem-solving, future planning, intellectual growth, self-reflection and creativity, emotional rejuvenation, and relaxation [9,32]. Given the wide range of advantages and benefits associated with positive solitude, we suggest that exploring its moderating influence on the relationship between the 24 character strengths and flourishing could illuminate the nuanced interplay among individual strengths, positive solitude, and flourishing in later life.

### 1.4. The Current Study

The current study aims to deepen the existing knowledge regarding the associations linking two of the personal resources that contribute to resilience and wellbeing in the second half of life: the 24 character strengths and the skill of positive solitude [9,20,31,48]. We hypothesized that (H1) participants with higher levels of the 24 character strengths will demonstrate higher levels of flourishing, (H2) that participants with higher levels of positive solitude will demonstrate higher levels of flourishing, and that (H3) positive solitude will moderate the association between the 24 character strengths and flourishing. Since there is no literature regarding the relationships between the variables examined in H3, the direction of the moderation was tentatively tested, and this hypothesis was framed as an exploratory analysis.

## 2. Method

### 2.1. Participants and Procedure

This study utilized data from a convenience sample via a free online cross-sectional survey conducted in 2022. Participants included community-dwelling adults from 47 countries worldwide who engaged with the study through the Qualtrics web platform. Out of the 10,788 participants who accessed the survey, 9703 participants under the age of 50 were excluded from the analysis. The final sample comprised 1085 participants, with a mean age of 57.20 years (SD = 6.24; age range = 50–87). Notably, 71.5% of the participants were women; the majority were married (64.7%) and had attained higher education (93.7%). Ethical approval for the study was obtained in August 2023 by the departmental ethical review board (IRB) at the last author’s university (number 06-23).

### 2.2. Measures

Socio-demographic characteristics questionnaire: These included age, gender (categorized into 1 = male, 2 = female), marital status (categorized into 0 = single, divorced or widowed; 1 = married or cohabiting), and education (0 = Less than a high school degree, 1 = high school degree or GED, 2 = some college but no degree, 3 = certificate or technical degree, 4 = Some graduate or professional school, 5 = Associate’s degree, 6 = Bachelor’s degree, and 7 = completed Master’s, Doctorate, or Professional degree (post-Bachelor’s)).

Flourishing was measured by the flourishing scale [49], which is rated on a 7-point Likert scale (ranging from 1 = strongly disagree to 7 = strongly agree), reflecting the degree of agreement with eight statements. Each statement refers to one of the following categories: good relationships, autonomy, a sense of self-efficacy, a sense of purpose, and positive feelings related to enjoyment, involvement, and life satisfaction. The items include statements such as “I lead a purposeful and meaningful life” and “I am a good person and live a good life”. An overall score is obtained by averaging the eight items [49]. A high score represents higher psychological resources and strengths [49]. The internal reliability was high (Cronbach’s α = 0.89).

The *24* character strengths were measured by the VIA inventory of strengths (VIA–IS; 6). The VIA–IS is a 96-item self-report questionnaire that uses a 5-point Likert scale to measure the degree to which respondents agree that they endorse each of the 24 character strengths. An overall score is obtained by averaging the items [6]. The internal reliability was high (Cronbach’s α = 0.91).

The positive solitude questionnaire [47] was utilized to assess the skill of positive solitude. This new instrument is based on the semantics of the theoretical conceptualization for positive solitude. This 9-item questionnaire evaluates the degree to which individuals willingly prefer solitary experiences they view as enjoying. For instance, “finding time for myself contributes to my quality of life”. Participants rated their level of agreement with each item on a 5-point Likert scale, with response options ranging from 1 = “strongly disagree” to 5 = “completely agree”. The overall score is calculated as the mean of all items, with higher scores indicating better positive solitude skills. The reliability of the questionnaire was high, with a Cronbach’s α = 0.89.

### 2.3. Data Analysis

To test the study’s hypotheses, we conducted statistical analyses using SPSS software, version 27. Significant interactions were analyzed by Model 1 of simple moderation, utilizing the PROCESS 3.4 macro for SPSS [50]. Descriptive statistics and preliminary correlations among the study variables were computed.

Using hierarchical linear regression, the study hypotheses were examined. For hypotheses 1 and 2, demographic variables and covariates (age, gender, education, and marital status) were entered in the first step. In the second step, 24 character strengths and the skill of positive solitude were entered. Finally, in the third step, to examine the third hypothesis, the effect of the interaction between 24 character strengths and the skill of positive solitude on flourishing was tested, while before the analyses, continuous predictors were mean centered. A preliminary analysis was also conducted to assess potential multicollinearity. The results, with tolerance rates ranging from 0.86 to 0.99 and a VIF of 1.00–1.16 for the study variables, confirmed the absence of any multicollinearity concerns [51].

## 3. Results

Table 1 presents the descriptive statistics of the study variables. As seen in Table 1, a significant strong positive correlation was found between the 24 character strengths (on the general scale) and flourishing (*r =* 0.61; *p* < 0.001) and a moderate positive correlation was found between the skill of positive solitude and flourishing (*r =* 0.29; *p* < 0.001). Additionally, a significant positive moderate correlation (*r =* 0.31; *p* < 0.001) was found between the 24 character strengths on the general scale and the skill of positive solitude. A low but significant positive correlation was found between years of education and flourishing, as well as between marital status and flourishing, indicating that flourishing tends to be slightly more common among individuals with higher education (*r* = 0.10; *p* < 0.001) and among married couples (*r* = 0.14; *p* < 0.001) (for further information see Table 1).

Furthermore, an examination of the correlations between each of the 24 character strengths and the skill of positive solitude reveals significant moderate positive correlations for strengths such as appreciation of beauty and excellence (*r =* 0.38; *p* < 0.001), love of learning (*r* = 0.33; *p* < 0.001), curiosity and hope (*r* = 0.30; *p* < 0.001), creativity, gratitude (*r =* 0.26; *p* < 0.001) and perspective (*r =* 0.25; *p* < 0.001). Conversely, non-significant or extremely low positive correlations were observed for strengths such as love, kindness, and teamwork.

Next, and following our hypotheses, we conducted a hierarchical linear regression to examine the relationships between the 24 character strengths, the skill of positive solitude, and flourishing (see Table 2). After controlling demographics in step 1 (age, gender, education, marital status), the 24 character strengths and the skill of positive solitude were entered in the second step. Following hypotheses 1 and 2, participants who reported higher 24 character strengths (β = 0.58, *p* < 0.001) and better positive solitude skills (β = 0.10, *p* < 0.001) reported higher flourishing level.

Finally, in line with the third hypothesis, a significant interaction was found between the 24 character strengths and the skill of positive solitude, who were entered in Step 3, which accounted for an additional 1% of the variance of flourishing (β *=* −0.102, *p* < 0.001). The overall model explained 41% of the variance in flourishing.

**Figure 1:**
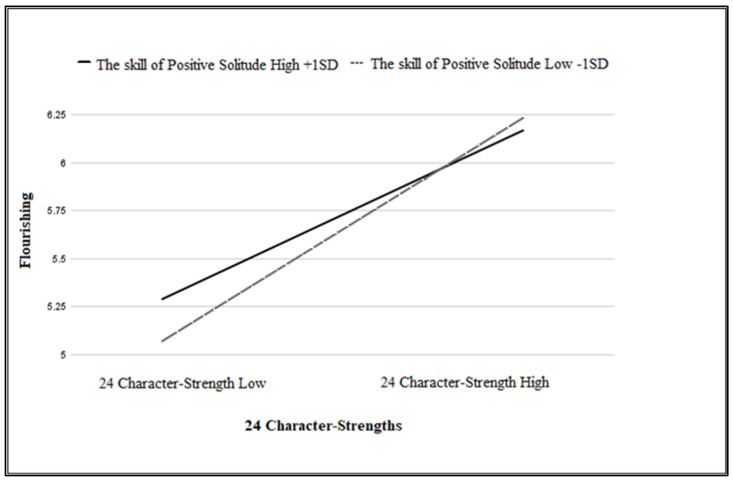
The skill of positive solitude moderates the relationship between the 24 character strengths and flourishing.

To probe the significant interaction PROCESS 3.4 macro software was used [50]. Figure 1 presents the two-way interaction between the skill of positive solitude and the 24 character strengths on flourishing. As can be seen, for participants who demonstrated low positive solitude skills (−1SD), the relationship between the 24 character strengths and flourishing was significant and positive (β = 1.51, *p* > 0.001), while for participants who demonstrated high levels of positive solitude skills (+1SD), the relationship between the 24 character strengths and flourishing was weaker, although positive and significant (β = 1.11, *p* < 0.001).

## 4. Discussion

This study examined the association between flourishing and two valuable internal resources: the 24 character strengths and the skill of positive solitude in the second half of life. The findings supported the proposed hypotheses.

The first hypothesis was supported by the findings, and this is in line with previous studies, which demonstrated the contribution of the 24 character strengths to mental and psychological wellbeing during middle and late adulthood [6,17,52].

In line with the second hypothesis, the positive association between positive solitude and flourishing corresponds well with current findings regarding the relationship between positive solitude and wellbeing in later life [9,53]. This relationship may indicate that individuals who possess strong positive solitude skills are better equipped to harness their inner resources for the improvement of their overall wellbeing [11].

Consistent with the third hypothesis, positive solitude moderated the relationship between the 24 character strengths and flourishing. This moderation demonstrated that, while the positive relationship between the 24 character strengths and flourishing was highly significant among those with lower positive solitude skills, it was much weaker, although still significant, among those with higher positive solitude skills. The moderating effect of positive solitude on the relationship between the 24 character strengths and flourishing becomes particularly significant in mid and later life, due to the shrinking social connections. This moderation highlights the unique interplay between character strengths and positive solitude, suggesting that when character strengths are low, higher levels of positive solitude compensate for the shortage in character strengths, to maintain sufficient flourishing. This moderation effect may hint at the importance of not only possessing individual 24 character strengths but also of being able to cultivate positive solitude skills which may compensate as an alternative personal resource to the 24 character strengths. This interpretation adds to the value of developing and nurturing the skill of positive solitude, alongside the 24 character strengths.

While this study offers further evidence of the importance of positive solitude as a skill, several limitations should be acknowledged. First, the cross-sectional design restricts the ability to determine causality between the variables. Therefore, caution is warranted when interpreting these findings in terms of cause and effect. Future longitudinal studies can examine causal relationships between the variables. Additionally, this study is based on self-report scales, which are subjected to social desirability. Finally, although the sample included participants from 47 countries, data collection occurred primarily via the internet in the U.S., potentially introducing a cultural bias reflective of an educated Western society and gender bound, as more women answered the questionnaires. The individualistic cultural values prevalent in this context are closely related to the positive solitude conceptions and may not adequately capture the perspectives of individuals from non-Western collectivist backgrounds. Therefore, caution is warranted when generalizing these findings beyond the studied population. Future research should aim to incorporate a broader range of cultural and demographic contexts to enhance the generalizability of the results.

Nevertheless, this research provides evidence for the relevance of the skill of positive solitude to flourishing in the second half of life, as well as initial insights regarding the relationship between this ability and the 24 character strengths. The correlations that were found in this study provide a first hint that in the second half of life, the skill of positive solitude is associated with specific personality strengths such as a love of learning and appreciation for beauty and excellence. Future studies may delve into this relationship and aim to deepen our theoretical understanding of the qualities of the skill of positive solitude. Understanding the qualities can pave the way for developing intervention strategies by which to promote the skill of positive solitude during late life. Such interventions may also aim at improving important tendencies, such as love for learning, and appreciation of beauty and excellence, that do not diminish throughout the years. Thus, the study contributes to the growing body of literature on positive solitude skills and the ways they can be enhanced in the second half of life.

## Figures and Tables

**Table 1:** Descriptive statistics and correlations of the study variables (N = 1085).

	M/%	SD	1	2	3	4	5	6	7
1. Age	57.20	6.24	1						
2. Gender	71.5%	-	−0.02	1					
3. Education	5.26	2.03	0.02	−0.02	1				
4. Marital Status	64.7%	-	−0.10 ***	−0.09 ***	0.01	1			
5. 24 character strengths	3.76	0.40	0.10 ***	−0.02	0.05	0.02	1		
6. Flourishing	5.70	0.90	0.03	0.05	0.10 ***	0.14 ***	0.61 ***	1	
7. The skill of positive Solitude	3.85	0.74	−0.01	0.08 ***	0.17 ***	−0.06	0.31 ***	0.29 ***	1

*** *p* < 0.001.

**Table 2:** Linear regression analysis examining the relationship between the skill of positive solitude and the 24 character strengths on flourishing (N = 1080).

Model Variables		B	SE	β	F	R^2^	R^2^Δ
Step 1					9.27 ***	0.03 ***	0.03
	Age	−0.001	0.004	0.05			
	Gender	0.13	0.06	0.07 *			
	Education	0.05	0.01	0.10 ***			
	Marital Status	0.28	0.06	0.14 ***			
Step 2					123.56 ***	0.41 ***	0.37
	24 Character strengths	1.32	0.06	0.57 ***			
	The skill of Positive Solitude	0.12	0.03	0.09 ***			
Step 3					110.43 ***	0.42 ***	0.01
	24 Character strengths X the skill of Positive Solitude	−0.26	0.06	−0.10 ***			

* *p* < 0.05, *** *p* < 0.001.

## Data Availability

Restrictions apply to the availability of these data. Data were obtained from the VIA Institute and are available from the authors with the permission of the VIA Institute.
